# Work-related experiences and needs while undergoing curative cancer therapy: exploring the target population’s perspective during the developmental phase of a work-oriented intervention

**DOI:** 10.1007/s00520-025-09624-1

**Published:** 2025-06-17

**Authors:** Linda Eklund, Maria Engström, Angela G.E.M de Boer, Ulrika Bejerholm, Maria Fjell, Per Fessé, Sven Trygged, Anna Efverman

**Affiliations:** 1 Department of Caring Science, Faculty of Health and Occupational Studies, University of Gävle, Gävle, Sweden; 2 Department of Occupational Therapy and Healthcare Counselling, Region Dalarna, Ludvika, Sweden; 3 Amsterdam UMC, University of Amsterdam, Public and Occupational Health, Amsterdam, the Netherlands; 4 Department of Health Sciences, Medical Faculty, Lund University, Lund, Sweden; 5 Department of Psychiatry, Skåne University Hospital, Lund, Sweden; 6 Department of Neurobiology, Care Sciences and Society, Karolinska Institutet, Stockholm, Sweden; 7 Centre for Research and Development, Uppsala University/Region Gävleborg, Gävle, Sweden; 8 Department of Social Work, Criminology and Public Health Sciences, Faculty of Health and Occupational Studies, University of Gävle, Gävle, Sweden

**Keywords:** Cancer rehabilitation, Complex intervention, Occupational health, Oncology, Work-oriented intervention, Work-life balance

## Abstract

**Purpose:**

Cancer affects a great number of people during working life, and tailored interventions targeting this population are important. The present study, focused on the developmental phase of a work-oriented intervention, aimed to describe work-related experiences and needs among people undergoing curative cancer therapy.

**Methods:**

People (*n* = 22, age md 55 years, range 39–64, purposive sampling) undergoing adjuvant or neo-adjuvant chemo-/radiotherapy for breast (*n* = 12), prostate (*n* = 5), or colorectal (*n* = 5) cancer were individually interviewed using a semi-structured interview guide covering their ability to work and work-related facilitators, barriers, and needs. Qualitative content analysis with an inductive approach was applied.

**Results:**

When undergoing curative cancer treatment, striving for work–life balance and a normal life is challenging, but necessary for wellbeing, according to the participants. Five subthemes described their experiences and needs: “Cancer is not my identity, and working helps me experience a sense of self,” “I mostly have confidence in my future ability to work,” “I need to find a new balance between work, private life, and my changed health needs,” “Having flexible working conditions helps me work, and both strengthens and limits my wellbeing,” and “Having access to individual support, in which others and I participate, affects my ability to work.”

**Conclusion:**

While undergoing curative cancer therapy, striving for work–life balance and a normal life is challenging but necessary for wellbeing. Our study findings suggest that a work-oriented intervention tailored to individual needs, flexibility in working conditions, and cooperation between the employee and various stakeholders are warranted during the early stage of cancer therapy.

## Introduction

Cancer affects a great number of people during working life, and thus, work-oriented interventions, tailored to the target population, are important [1, 2]. Over the past 30 years, the incidence of cancer in people younger than 50 years of age has increased by 79 percentage points [3]. Advances in cancer therapies have significantly improved survival rates, thereby increasing the demand for effective rehabilitation [4]. The present demographic, social, and economic challenges require that society provide support to help people remain active in the labor market [5].

Being diagnosed with cancer and undergoing cancer therapies often limits people’s ability in daily and working life [6, 7]. About a quarter of cancer survivors had not returned to paid work after 2 to 14 years [2]. Seven years after a cancer diagnosis, 14% were permanently excluded from work participation [8]. Studies have shown that sickness absence is associated with decreased quality of life [4] and has a negative impact on dignity, social roles in family and society, and economic livelihood [9, 10]. Cancer rehabilitation strives to prevent and alleviate cancer-related consequences [11], and work-oriented interventions specifically address work-related consequences [2]. Previous work-oriented interventions have primarily been conducted after people have completed cancer therapy [7]. A recent Cochrane review identified 15 high-quality trials in this field, only a few of which addressed needs during early curative cancer therapy [2]. Interviews with people who had not returned to work after completing cancer therapy have highlighted the lack of work-related communication and support during an earlier stage [12–14]. Employees who successfully returned to work after completing their cancer therapy perceived early work-oriented support to be crucial to their return to work [15]. These converging findings highlight the importance of studying work-related experiences and needs at an early stage.

Because the social system influences return to work [1, 16], the target population’s work-related experiences and needs are important to address when developing work-oriented interventions [17]. The intervention should be tailored to each country’s social system [1], addressing needs, barriers, and facilitators, the goal being to increase the likelihood of successful implementation in practice [18]. Preceding the development of an early-stage work-oriented intervention, the aim of the present study was to describe the work-related experiences and needs of people undergoing curative cancer therapy.

## Methods

### Design and context

A descriptive, inductive qualitative interview study [19] involving individuals undergoing curative cancer therapy was conducted, contributing to the development phase of the Medical Research Council’s framework for complex interventions [17]. The Swedish Ethical Review Authority approved the study (2022–03686-01), and the participants gave their written informed consent. To ensure the trustworthiness, the Consolidated Criteria for Reporting Qualitative Studies (COREQ) checklist [20] was used to guide both the execution and reporting of the study.

Cancer care in Sweden is taxpayer funded and delivered by 21 collaborating healthcare regions, consisting of vast rural areas in addition to towns and cities, i.e., people often travel long distances to healthcare clinics. Physicians issue a medical sickness certification (25–100%) based on work ability and work demands. The employer (first 14 days), followed by the Social Insurance Agency (no absolute time restriction), is responsible for sickness pay [21].

### The study participants

**Fig. 1:**
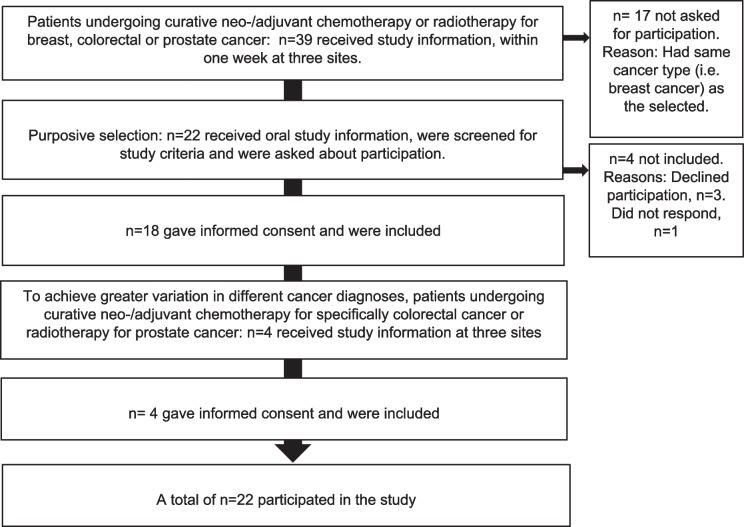
Inclusion of participants. Nurses within standard care delivered the written study information. The researchers (the first, fifth and sixth author) then orally summarized the study information over the telephone and asked for participants’ informed consent

We purposively recruited 22 participants (Fig. 1), who were informed about the study orally and in writing when they attended their ordinary treating oncology outpatient care settings. These were located in three healthcare regions in central Sweden, consisting of small- to middle-sized cities and rural areas, covering local, regional, and a university hospital. The inclusion criteria were people aged 18 to 64 years; employed/self-employed; receiving curative chemo-/radiotherapy for breast, prostate, or colorectal cancer; and who had the physical, mental, and linguistic capacity to give their informed consent. The purposive inclusion strived for variation in cancer types and regions (Table 1), which was believed to provide variation in work-related experiences and needs.

**Table 1:** Descriptive characteristics of the participants

Characteristics	Participants, *n* = 22
***Sociodemographic characteristics***	
**Age in years**, md, 25 th–75 th percentile	55, 49–59
Sex, *n* (%)	
Men	7 (32)
Women	15 (68)
**Civil status**, *n* (%)	
Married/living with partner	16 (73)
Living alone but has a partner	4 (18)
Living alone, has no partner	2 (9)
**Had children in their household**, *n* (%)	10 (45)
**Living condition**, *n* (%)	
Big city	1 (5)
Mid-size city	10 (45)
Small city	5 (23)
Small town/countryside	6 (27)
**Education level**^**a**^, *n* (%)	
Secondary school or occupational education	11 (50)
University	11 (50)
***Work-related characteristics***	
**Employment status,** *n* (%)	
Employed	20 (91)
Self-employed	2 (9)
**Percentage of sickness absence, *n* (%)**	
** 100%**	8 (36)
** 75%**	2 (9)
** 50%**	1 (5)
** 25%**	1 (5)
** 0%**	10 (45)
**Occupational sector**^**b**^	
Education, care or services	7 (32)
Technologies or engineering	2 (9)
Management or entrepreneurship	6 (27)
Other occupations	7 (32)
***Clinical characteristics***	
**Cancer type,** *n* (%**)**	
Breast	12 (54)
Prostate	5 (23)
Colorectal	5 (23)
**Months since cancer diagnosis**, md, 25 th–75 th percentile, (range)^**c**^	6, 3–7 (2–88)
**Received cancer therapy (neo-adjuvant or adjuvant) at the time of interview**, *n* (%)	
Neo-adjuvant	6 (27)
Adjuvant	16 (73)
**Received cancer therapies preceding interview**, *n* (%)	
Chemotherapy	5 (23)
Chemotherapy and radiotherapy	7 (32)
Radiotherapy^**d**^	10 (45)
Hormonal or antihormonal therapy added to chemoradiation	9 (41)
**Had comorbiditiese**, *n* (%)	8(36)

^a^None had just elementary education. Occupational education was not at the university level

^b^Presents categorized free-text responses

^c^Two participants had cancer for the second time; time since first diagnosis is presented

^d^One participant received both external and internal radiotherapy

^e^The comorbidities were fibromyalgia, chronic pain, myocardial infarction, herniated disc, exhaustion disorder. Md, median value

According to responses (digitally or using pen and paper) to previously applied study-specific descriptive questions relevant for work participation [6], the participants were 39 to 64 years of age, on sickness absence 0 to 100%, and their occupations represent the private and public sector (Table 1).

### Data collection

Three interviewers, L.E. (woman, registered occupational therapist), M.F. (woman, oncology nurse), and P.F. (male, oncology nurse) all experienced in cancer care (20–24 years of professional experience), individually interviewed the participants; this occurred independent of their care. The interviewers had limited (L.E.) or extensive (P.F. and M.F.) experience in qualitative interviewing. All interviews [19] followed a semi-structured interview guide (Table 2), developed by the authors based on previous research [2, 22], which informed the selection of relevant topics to address in relation to the aim. Interviews were audio recorded and performed in a place chosen by the participants, mostly in their home, at the clinic, or digitally (about a third). The interviews ranged from 16 to 62 min (median 41 min) and were transcribed verbatim. The first interviews (two each) were considered pilot interviews and were discussed among the interviewers, not resulting in any changes and thus included.

**Table 2:** The semi-structured interview guide

**Interview questions**^a^
1. Can you describe your current work situation?
2. How do you feel your wellbeing is affected based on your current work situation?^b^
3. What facilitates or hinders your work situation?^c^
4. What are your thoughts about your chance of being able to return to (/stay in) work in the long run?
5. Can you describe the current support you are receiving related to your wellbeing in your current work situation?^b^
6. Based on your perceptions, how should support/interventions promoting work participation and wellbeing at work in people with cancer be designed?

The interviewer applied a code number replacing the participant’s name during the interview

^a^General follow-up questions included: “Can you tell me more?” and “What do you mean?”

^b^Regardless of working or being on sickness absence

^c^This question covered the follow-up questions on what facilitates and what impairs work participation and work-related wellbeing

### Data analysis

The first author listened to the interviews, read the transcripts repeatedly, and conducted an inductive qualitative content analysis [23] in collaboration with the second author. The content addressing the study aim was divided into meaning units of suitable length, so that the context could still be understood during condensation and coding. During the coding process, the analyzers stayed as close to the manifest content as possible, to avoid their pre-understanding having any impact. Codes were compared for similarities and dissimilarities and were sorted into subthemes, involving interpretation of the latent content [24] of the text. For example, codes like “*working helps me keep my normal routines*,” and “*wanted to be treated as usual*,” both contributed to the subtheme “*Cancer is not my identity, and work helps me experience a sense of self*.” Discussions and interpretations regarding the overall content resulted in one main theme. The analysis process moved back and forth between the analysis steps. The main analyzer, L.E., discussed the above-mentioned steps with M.E., reaching consensus on interpretation, coding, and thematization. L.E., M.E., and the last author, A.E., then wrote a descriptive story for each subtheme, illustrated using quotations; see Table 3. Then, employing triangulation, the fifth author, S.E., read the transcripts, compared them with the proposed themes, and confirmed that the analysis was trustworthy [25]. All authors read and discussed the thematization to reach a consensus, leading to minor linguistic adjustments of the final themes and labeling.

**Table 3:** Work-related experiences and needs illustrative quotations representing the five subthemes

Subtheme	Phrases from results	Quotations
Subtheme 1: cancer is not my identity, and work helps experience a sense of self	The participants who worked described work as helping them experience a sense of self and not always identify as a cancer patient Due to individual preferences in boundaries between private roles and roles in working life, some participants wanted to focus on work tasks and a professional dialogue, as this approach held at bay the feeling of being “the cancer patient” at work. That kind of stigma increased the psychological burden They all valued being able to maintain their normal roles, such as being a colleague, a professional…	*“It’s nice to work, otherwise it’s easy to get pulled down into thoughts about having cancer”* (participant 1) *“I was very, very clear about them treating me like normal, otherwise I wouldn’t have been able work”* (participant 2) “I got a wig, otherwise I was a living neon sign and needed to talk about the disease at work meetings or with patients, which I couldn’t bear” (participant 3) *“I want to do my usual working tasks, do what I typically do. I want to start working again as soon as possible.”* (participant 4)
Subtheme 2: I feel mostly confident in future work ability	They all expected work participation to have a positive effect on their own and their family’s wellbeing in the future. However, worries about a cancer relapse, cancer-related consequences, e.g., incontinence, physical functioning limitations, and restrictions after tumor surgery, or uncertainty concerning how to manage with, e.g., their stoma, were problems they felt might affect their future work ability Experienced self-perceived overall health affected participants’ confidence or lack of confidence in their future work ability. However, they had mostly positive beliefs regarding being part of an active future working life	*“As long as I don't have any major side effects I only see working as a positive. But if I have trouble with incontinence, I don’t know.”* (participant 5) *“I don't know how hard this extreme irradiation is. Nothing you feel right away. Something that will show up a little further down the line.”* (participant 6) *“I think if I get better working conditions, and get some solid ground under my feet, then this could be really good. I have to work on my self-confidence as well. So, I want to believe, I really want to believe that it’s going to turn out really well!”* (participant 7)
Subtheme 3: I need finding a new balance between work, personal life and changed health needs	The amount of work vs. sick leave and the demands of work and everyday life needed to be tailored to the new life situation so as not to risk diminished wellbeing. Otherwise, working led to an excessive load and caused fatigue The cancer therapy was time-consuming and sometimes took place in other cities, which meant traveling and overnight stays. Enabling work under these circumstances was compared to a challenging balancing act The desire to prioritize family time and social contacts to promote psychological wellbeing and manage their increasing health needs was central to several of the participants	*“You need to have some discipline so it doesn’t end up being too much as well, putting limits on yourself can be difficult”* (participant 8) *“It’s taken a lot of time going to the hospital, it’s like working half time. Different treatment times every day, tried to adapt treatment times and really tried to get it together, it hasn’t been easy.”* (participant 9) “*For me, getting a break from work has been good. In my situation, I will probably re-evaluate a little, some things may not be so important. Family and health will be first.”* (participant 10)
Subtheme 4: flexibility in work conditions is an enabler to work but also a risk factor for wellbeing	Participants working in organizations with more flexibility regarding working hours, replacement systems, working tasks and work location experienced greater possibilities to work during ongoing cancer therapy. Being able to control working hours and tasks in relation to the therapy schedule and the day-to-day condition created wellbeing and facilitated work participation Overall, sometimes the participants’ descriptions of flexibility were associated with negative consequences for perceived health and wellbeing, because they pushed themselves too hard in their working situation. Flexible solutions affected their decisions in that they requested less sickness absence than they actually needed, and they discovered too late that their mental and physical capacity were not sufficient in relation to the demands of their cancer therapy and work	*“I can work whenever I can and when it’s right for me. No one is dependent in my work”* (participant 11) *“I received computer screens to my home making it possible for me to work whenever I want and when I feel my wellbeing allows it”* (participant 12) *“At the same time, working from home made it very difficult to maybe draw that line and be on sick leave instead, because it’s been so accessible.”* (participant 9) *“I was allowed to plan my working hours freely with my employer and then we planned for two longer working days per week instead of working shorter every day. It didn’t turn out well as I was completely drained out of energy” (participant 13)*
Subtheme 5: available individual support, where I and others participate, affects my possibility to work	Lack of knowledge regarding available support, obligations and current regulations for sickness absence hindered work and made the situation stressful Their employer, colleagues and healthcare were important in facilitating work. They were creating a welcoming feeling at the workplace, providing information about healthcare interventions, how to manage symptoms, and adapting working conditions and sickness certificates if needed Being left alone with work planning was frustrating and created a sense of loneliness and stress Investigating participants’ needs and providing individualized support improved the possibilities for work participation The degree to which family members provided emotional support and lessened household burdens affected participants’ opportunities for rest or self-care, thus modifying their ability to work in a positive or a negative direction	*“I don’t know what my employer is required to help me with and stuff like that*.*”* (participant 6) *“If it’s long-lasting, and then you have meetings like this together, maybe a relative can take part. It’s great when everyone hears what everyone is saying”* (participant 3) *“Got the news that it’s probably cancer, they said you can do what you want, if you want to work or not, and then you were left alone with it”* (participant 14) *“The healthcare system and the workplace should have more contact. I have the problems I have. Another person may have completely different problems. Some need more and others need less. They should have some kind of idea about what it should be like”* (participant 7) *“My husband helps we with the cooking in the evening so I can rest after work and get new energy” (participant 3)*

The sub-themes, result phrases, and quotations from the interviews are described

## Results

**Fig. 2:**
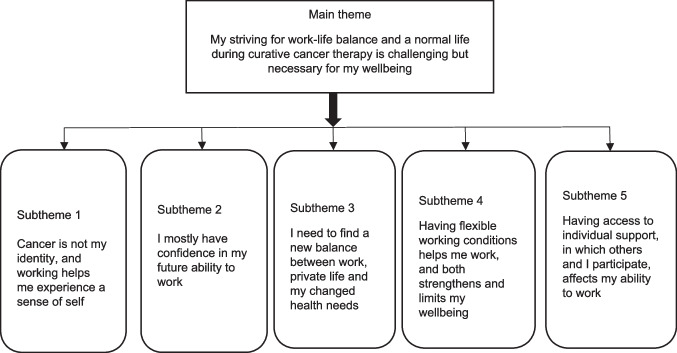
Main theme and subthemes describing work-related experiences and needs among people undergoing curative cancer therapy

The analysis and interpretation of the participants’ work-related experiences and needs resulted in one main theme “My striving for work–life balance and a normal life during curative cancer therapy is challenging but necessary for my wellbeing.” The descriptions concerned five subthemes (Fig. 2 and Table 3).

### Subtheme 1: Cancer is not my identity, and working helps me experience a sense of self

The participants who worked described work as helping them experience a sense of self and not always identify as a cancer patient. They all valued being able to maintain their normal roles, such as being a colleague, a professional, and there were descriptions of the importance of contributing to the family’s livelihood while undergoing cancer therapy. Some participants described a desire to maintain normal roles and routines, which led them to ‘secretly’ work despite being on full-time sick leave. Having social contact with the workplace created joy. The participants who worked to some degree appreciated using their skills and being useful and needed. They appreciated thoughtfulness on the part of colleagues, but due to individual preferences in boundaries between private roles and roles in working life, some of the participants wanted to focus on work tasks and a professional dialogue, as this approach held at bay the feeling of being “the cancer patient” at work. That kind of stigma increased the psychological burden.

### Subtheme 2: I mostly have confidence in my future ability to work

Although the participants described their work ability as lowered or non-existent while undergoing cancer therapy, they were mostly positive about their future work ability—with some reservations. They all expected work participation to have a positive effect on their own and their family’s wellbeing in the future. However, worries about a cancer relapse, cancer-related consequences, e.g., incontinence, physical functioning limitations, and restrictions after tumor surgery, or uncertainty concerning how to manage with, e.g., their stoma, were problems they felt might affect their future work ability. Further, experienced self-perceived overall health affected participants’ confidence or lack of confidence in their future work ability. However, they had mostly positive beliefs regarding being part of an active future working life. Having the opportunity to return to their previous workplace and having felt confident at the workplace prior to the cancer were perceived to have a positive effect on their confidence in being able to continue working or returning to work in the future.

### Subtheme 3: I need to find a new balance between work, private life, and my changed health needs

The participants reflected on the need to find a balance between work, personal life, and health-related needs while undergoing cancer therapy. Work was important in providing a distraction from the cancer diagnosis and therapy. At the same time, new needs and new priorities among activities in everyday life did emerge. The desire to prioritize family time and social contacts to promote psychological wellbeing and manage their increasing health needs was central to several of the participants. Managing health needs could involve, e.g., engaging in physical activity and taking time for their increased need for rest and sleep. The cancer therapy was time-consuming and sometimes took place in other cities, which meant traveling and overnight stays. Enabling work under these circumstances was compared to a challenging balancing act. The amount of work vs. sick leave and the demands of work and everyday life needed to be tailored to the new life situation so as not to risk diminished wellbeing. Otherwise, working led to an excessive load and caused fatigue. The cancer therapy had caused loss of energy and a need for more rest. A lack of mental energy was experienced when trying to find a new balance, because difficulties with concentration, memory, and communicating with many people during the same workday impaired their work ability. Based on individual needs such as physical and mental wellbeing, occupation, type of therapy, and physical restrictions, working hours and need for sickness absence varied from full-time sickness absence to full-time work. In some cases, the stressful experience of having a reduced income forced participants to return to work. Working part-time, gradually increasing working hours, and having enough time to assess what was manageable after sickness absence were perceived to facilitate returning to work. However, part-time work was challenging, as it involved trying to limit working hours and to not perform more work than was reasonable; this was especially difficult because there was more fluctuation in their work ability than there was flexibility of their sickness certificates.

### Subtheme 4: Having flexible working conditions helps me work, and both strengthens and limits my wellbeing

There was great variation in the working conditions described due to the different occupations. Participants working in organizations with more flexibility regarding working hours, replacement systems, working tasks, and work location experienced greater possibilities to work during ongoing cancer therapy. Being able to control working hours and tasks in relation to the therapy schedule and the day-to-day condition created wellbeing and facilitated work participation. Digital work forums facilitated work by enabling work from another location and minimizing physical contact that could be exhausting and entail the risk of infection. Participants working in educational, care, or service occupations described limited flexibility and experienced difficulties avoiding contact with other people to reduce the risk of infection. They also mentioned the limited possibilities to take breaks as problematic owing to their experienced fatigue. Self-employed participants, one of whom also had a manager role, experienced that being able to work more flexibly when undergoing cancer treatment made work participation possible. At the same time, there were difficulties associated with being on sickness absence for self-employed individuals with manager roles, as employees needed support and personal economy was dependent on production. These demands forced the participant to work more than was good for wellbeing. Overall, sometimes the participants’ descriptions of flexibility were associated with negative consequences for perceived health and wellbeing because they pushed themselves too hard in their working situation. Flexible solutions affected their decisions in that they requested less sickness absence than they actually needed, and they discovered too late that their mental and physical capacity was not sufficient in relation to the demands of their cancer therapy and work.

### Subtheme 5: Having access to individual support, in which others and I participate, affects my ability to work

When the participants had access to individually adapted support, they perceived that work created self-efficacy and meaningfulness in everyday life and positively affected wellbeing. For those experiencing a lack of support and a high emotional/physical burden due to their cancer illness, the relation to work became stressful. Their employer, colleagues, and healthcare were important in facilitating work by creating a welcoming feeling at the workplace, providing information about healthcare interventions, how to manage symptoms, and adapting working conditions and sickness certificates if needed.

Lack of knowledge regarding available support, obligations, and current regulations for sickness absence hindered work and made the situation stressful. Their planning for return-to-work did not always match the Swedish Social Insurance Agency’s rules on working hours for part-time sickness absence, which became an aggravating circumstance. Due to the fluctuation in their wellbeing, the need for sickness absence sometimes varied from day to day. Their need for flexibility in relation to time was not in accordance with the Swedish Social Insurance Agency’s rules. Not being involved in work planning or, the opposite, being left alone with work planning was frustrating and created a sense of loneliness and stress. Full-time sickness certificates were sometimes issued routinely without discussing the participants’ non-communicated thoughts on, e.g., workplace adaptations or part-time sickness absence. Investigating participants’ needs and providing individualized support improved the possibilities for work participation. When the participants’ employer tried to create flexible adaptations and expressed appreciation, their work-related self-efficacy was strengthened. In some cases, specific solutions were needed to limit the risks associated with work in relation to cancer therapy. For example, participants with a peripherally inserted central catheter who worked in healthcare or social welfare highlighted concerns about accidental physical harm, prompting the need for risk mitigation strategies.

Not all participants but a few of them mentioned family members as important stakeholders. The degree to which family members provided emotional support and lessened household burdens affected participants’ opportunities for rest or self-care, thus modifying their ability to work in a positive or a negative direction.

## Discussion

The present study found that, according to the people undergoing curative cancer therapy, striving for work-life balance and a normal life was challenging but necessary for wellbeing. In line with holistic theoretical models on cancer and work [26], various kinds of needs, barriers and facilitating circumstances related to work and wellbeing were described.

The finding shows that work helped people undergoing cancer therapy experience a sense of self represented their striving to maintain their role identities as themselves and not as someone who is ill. They expressed a desire to be seen as a colleague, employee or employer. This is similar to findings from previous studies conducted after cancer therapy completion, where work was described as important to identity and social roles, even in cases of a long-term cancer disease [27, 28] and other diseases. When an individual’s work defines who he/she is, being on sickness absence may reduce wellbeing on an existential level and require finding a new role or “self” [29]. Our study participants managed the boundaries between the role of being a cancer patient and being an employee or colleague in different ways. Some preferred keeping a strict boundary between working roles and personal life. Work gave them an opportunity to be treated as themselves and as a professional. Others preferred an integrated boundary between roles in working and personal life. According to “Boundary Theory” [30], individuals have different preferences regarding the permeability of boundaries. The fit between individual preferences and everyday life demands regarding boundary management will affect our wellbeing. When people experience a new life situation, such as undergoing cancer therapy, boundary management strategies become relevant to coping with the new life situation and new boundary challenges [30].

The study participants mostly felt confident in their future work ability, despite some reservations regarding long-term cancer-related consequences. That seems promising, as self-efficacy plays an important role in facilitating the process of returning to work [16, 22] and may be important to strengthen when helping people with cancer manage the various cancer-related consequences in relation to work. People with cancer have previously reported that worrying about developing persistent cancer-related consequences is the most disturbing issue while undergoing cancer therapy [31]. Notably, the participants in the current study experienced good self-efficacy. Identifying individuals with low self-efficacy related to recovery and return to work should be a priority in future interventions. Furthermore, reducing fatigue and promoting physical activity [2, 6] may be beneficial. Regardless of the nature of the barriers experienced, a person-centered approach is essential in work-related cancer rehabilitation to support and maintain self-efficacy.

Discovering a new balance between work, everyday life, and changed health needs was experienced as necessary while undergoing cancer therapy, demonstrating the complexity of work participation. Not only working conditions but also the entire work-life balance must be considered if work participation and wellbeing are to be achieved [27], just as during sickness absence for other conditions [32]. Some needed a period of sickness absence to manage their new life situation, while others needed to work. There were descriptions of feeling forced to return to work for financial reasons or of fearing loss of clients or one’s career, especially when self-employed, as has been shown in previous research [33]. A third of people undergoing cancer therapy previously reported that, to ensure wellbeing, they would have needed more time off from work [28]. Even employees in countries with publicly funded healthcare and social welfare systems experience major social and economic consequences, given that long periods of sickness absence are associated with lower incomes and increased financial burden on households [34]. The participants called for support in decision-making regarding sickness absence versus work, because they hardly knew what was best in their new challenging situation. Due to, e.g., fatigue, they needed to find a new balance involving undergoing cancer therapy, associated travelling, caring for family, resting, engaging in physical activity, and work participation. Given that fatigue has been found to impair work ability while undergoing cancer therapy and even seven years afterwards [6, 35] as well as to strongly predict return-to-work outcomes [1, 22], it would seem important to provide support for fatigued employees. Fortunately, the study participants reported having made time to engage in physical activity in their new work-life balance. Systematic reviews have reported that engaging in physical activity while undergoing cancer therapy improves return-to-work outcomes [2, 36].

Flexible working conditions enabled work among participants working in sectors other than education, care or services. As in other long-term illnesses, work participation opportunities still seem to be dependent on social factors [37]. However, flexible working conditions were also associated with reduced wellbeing, as flexibility caused employees to work even though sickness absence would have been better for them. This was an interesting finding, because flexible working conditions have previously solely been described as facilitating work and wellbeing [22, 38]. Difficulties related to role boundaries and role conflicts [30] can occur during distance work. Strategies for dealing with personal boundary preferences may be important to experiencing wellbeing in the work situation. The employer and employee need to create conditions for a good working environment with clear requirements, otherwise there is a risk that extended working hours, lack of rest, social isolation, poor ergonomics, etc., will lead to worsened perceived health [39].

Support involving the affected employees and stakeholders, i.e., employers, healthcare professionals and family, was thought to affect work participation while undergoing cancer therapy. The participants, in line with “Social Support Theory” [40], described different kinds of support: emotional, social, practical, and informational. Their families either were promoters or constituted a barrier, depending on the amount of practical support, for example, sharing household work. Person-centered information and interventions, involving cooperation between stakeholders, were previously found to effectively promote work participation among people with cancer in countries other than Sweden [2, 26]. Participants in the present study experienced that person-centered emotional, social, practical, and informational support, involving collaboration between stakeholders, promoted adaptation to work-related issues while undergoing cancer therapy, thus improving work-related wellbeing. They described themselves, their employers and healthcare professionals, e.g., their oncologist, as playing important roles in this process. However, many oncologists find the dual role of being the patient’s physician and being the prescriber of sickness certificates to be problematic, because the latter role involves several stakeholders, e.g., employers [41], who also find this issue complex [42]. Problems of cooperation may lead to unnecessarily long sickness certificates among people with cancer [42, 43]. We believe that integrating the views of different working life stakeholders is important when developing work-oriented interventions, the goal being to overcome their barriers to contributing to and cooperating in work-oriented interventions.

We found the Consolidated Criteria for Reporting Qualitative Studies (COREQ) checklist [20] to be beneficial to ensure trustworthiness throughout the study. Through purposive sampling, we achieved variation in characteristics believed to give variation in experiences, e.g., different cancer types (among those most treated using chemoradiation) and regions. Further, the sample includes men and women, of various ages, employed in different occupational sectors, and undergoing various therapy combinations, which is believed to enrich the variety of perspectives and increase credibility [21]. The sample size of 22 participants was considered appropriate; in qualitative research, the richness of data is more important than the sample size per se [44]. The interviewers represented two healthcare occupations; all were experienced in cancer care/rehabilitation. To avoid L.E.’s limited experience in interviewing negatively affecting the quality of the interviews, a semi-structured interview guide with open-ended questions was used to ensure the dependability and richness of the interview content. Further, the three interviewers conducted two pilot interviews each and thoroughly discussed them to ensure quality. We found qualitative content analysis [23], which focuses on describing variation in experiences, to be suitable to the study aim. To maintain rigor and transparency, documentation of the coding process was detailed, and peer debriefing occurred between the main analyzers. For increased credibility, triangulation [25] of the content analysis in relation to the content of the transcripts was conducted. As suggested [20], examples of the analysis process and presentation of representative quotations were provided. Open inter-author discussions were helpful in maintaining judgements of similarities and differences of content over time. None of the main analyzers had a personal history of cancer, which made the analysis process open-minded. To help readers determine whether the results are transferable, we thoroughly described the context, participants’ characteristics, as well as data collection and data analysis, as previously suggested [20]. Transferability to other contexts, cancer types and stages is limited due to the qualitative design, and thus interviews also need to be conducted after completed cancer therapy. Rather many of the participants had comorbidities, 36 percent, which was however not more than was seen in another cancer cohort, 44 percent [6]. None of the participants had only an elementary education, which means the findings are less representative of low-educated people’s experiences [45]. One inclusion criterion was being employed/self-employed, which limits the relevance to unemployed people, who have a higher risk of long-term exclusion from work participation [45]. Because it was important to us to ensure autonomy by collecting informed consent, another limitation is that people who did not understand Swedish were excluded. This limits the study’s ability to address potential challenging experiences regarding language barriers in the work-oriented cancer rehabilitation journey.

The study gave us valuable information about work-related experiences and challenges that need to be addressed when developing [17, 18] a work-oriented intervention tailored to the needs. By increasing our knowledge of needs, facilitators and barriers, the study findings will enhance the likelihood that the intervention will be successfully implemented and put into practice [17, 18]. The need for person-centered rehabilitation efforts, participation and cooperation between stakeholders, as well as new insights into the potential risk of diminished wellbeing associated with working flexibly during ongoing cancer therapy are important to address in an intervention meant to achieve workplace participation and wellbeing. In future interventions, individual assessments will be needed to identify both barriers and facilitators to work participation, as well as the perceived pros and cons of working. Such assessments may support the decision-making process around returning to or remaining at work. Based on the findings from the current study, the research team’s next logical step is to develop a work-oriented intervention, drawing on previous [2, 22] and ongoing research. In line with the Medical Research Council’s framework [17], this intervention will then need to be evaluated in terms of its feasibility and effectiveness in supporting work participation and wellbeing.

To conclude, striving for a work-life balance and a normal life during curative cancer therapy was experienced as challenging but necessary for wellbeing. Since more support to deal with this challenge was asked for, this implies that work-oriented rehabilitation, tailored to individual needs, flexibility in working conditions and cooperation between the employee and various stakeholders are warranted during the early stage of curative cancer therapy. We recommend that future studies investigate the views of different stakeholders, and that work-oriented interventions be co-designed with both the target population and other stakeholders to better meet their needs.

## Data Availability

The data generated from this study are not shared openly to protect the privacy of study participants; confidential data might be available upon request to the corresponding author.
